# Clinical utility of comprehensive circulating tumor DNA genotyping compared with standard of care tissue testing in patients with newly diagnosed metastatic colorectal cancer

**DOI:** 10.1016/j.esmoop.2022.100481

**Published:** 2022-05-04

**Authors:** M. Benavides, J. Alcaide-Garcia, E. Torres, S. Gil-Calle, I. Sevilla, R. Wolman, G. Durán, M. Álvarez, C. Reyna-Fortes, I. Ales, T. Pereda, M. Robles, M. Kushnir, J. Odegaard, I. Faull, E. Alba

**Affiliations:** 1 Medical Oncology Intercenter Unit, Hospital Universitario Regional y Virgen de la Victoria, IBIMA, Málaga, Spain; 2 Medical Oncology Department, Hospital Costa del Sol, IBIMA, Málaga, Spain; 3 Medical Oncology Service, Hospital Xanit, Málaga, Spain; 4 Medical Oncology Department, Hospital Universitario San Cecilio, Granada, Spain; 5 Cancer Molecular Biology Laboratory (CIMES), University of Málaga, Málaga, Spain; 6 Pathology Department, Hospital Costa del Sol, IBIMA, Marbella, Spain; 7 Medical Oncology Department, Hospital Costa del Sol, Marbella, Spain; 8 Guardant Health Inc, Redwood, USA; 9 Medical Oncology Intercenter Unit, Hospital Universitario Regional y Virgen de la Victoria, IBIMA, CIBERONC, Málaga, Spain

**Keywords:** metastatic colorectal cancer, circulating tumor DNA, liquid biopsy, biomarker, next-generation sequencing, genomic profiling

## Abstract

**Background:**

Comprehensive biomarker testing is essential in selecting optimal treatment for patients with metastatic colorectal cancer (mCRC); however, incomplete genotyping is widespread, with most patients not receiving testing for all guideline-recommended biomarkers, in part due to reliance on burdensome sequential tissue-based single-biomarker tests with long waiting times or availability of only archival tissue samples. We aimed to demonstrate that liquid biopsy, associated with rapid turnaround time (TAT) and lower patient burden, effectively identifies guideline-recommended biomarkers in mCRC relative to standard of care (SOC) tissue testing.

**Patients and methods:**

Prospectively enrolled patients with previously untreated mCRC undergoing physician discretion SOC tissue genotyping submitted pretreatment blood samples for comprehensive circulating tumor DNA (ctDNA) analysis with Guardant360 and targeted *RAS* and *BRAF* analysis with OncoBEAM.

**Results:**

Among 155 patients, physician discretion SOC tissue genotyping identified a guideline-recommended biomarker in 82 patients, versus 88 identified with comprehensive ctDNA (52.9% versus 56.8%, noninferiority demonstrated down to α = 0.005) and 69 identified with targeted PCR ctDNA analysis (52.9% versus 44.5%, noninferiority rejected at α = 0.05). Utilizing ctDNA in addition to tissue increased patient identification for a guideline-recommended biomarker by 19.5% by rescuing those without tissue results either due to tissue insufficiency, test failure, or false negatives. ctDNA median TAT was significantly faster than tissue testing when the complete process from sample acquisition to results was considered (median 10 versus 27 days, *P* < 0.0001), resulting in accelerated biomarker discovery, with 52.0% biomarker-positive patients identified by ctDNA versus 10.2% by SOC tissue 10 days after sample collection (*P* < 0.0001).

**Conclusions:**

Comprehensive ctDNA genotyping accurately identifies guideline-recommended biomarkers in patients with mCRC at a rate at least as high as SOC tissue genotyping, in a much shorter time. Based on these findings, the addition of ctDNA genotyping to clinical practice has significant potential to improve the care of patients with mCRC.

## Highlights

• Complete genotyping in mCRC is essential in the selection of optimal therapy in first line.
• Adoption of ctDNA testing into clinical practice has the potential to improve the care of patients with mCRC.
• Testing with ctDNA can identify biomarkers in a significantly shorter TAT than SOC tissue.
• Use of validated, high-sensitivity comprehensive ctDNA testing allows fully informed treatment decisions to be made earlier.
• Implementing ctDNA testing ahead of tissue can improve time to treatment initiation without compromising biomarker discovery.

## Introduction

Biomarker testing in patients with newly diagnosed metastatic colorectal cancer (mCRC) is crucial in the selection of first-line (1L) therapy. The National Comprehensive Cancer Network (NCCN) guidelines, the European Society for Medical Oncology (ESMO) recommendations for use of next-generation sequencing (NGS), and the Pan-Asian adapted ESMO consensus guidelines recommend testing for *KRAS, NRAS*, and *BRAF* V600E mutations as part of work-up for suspected, or confirmed, mCRC, while testing for microsatellite instability (MSI) is recommended for all patients with colorectal cancer, including those with early-stage disease.^1^, ^2^, ^3^, ^4^, ^5^ NCCN guidelines also recommend testing for *ERBB2* amplification^1^ and ESMO NGS recommendations refer to significant response to dual blockade in patients with *ERBB2*-positive mCRC demonstrated in clinical trials.^4^ On the ESMO Scale for Clinical Actionability of Molecular Targets (ESCAT), *BRAF* V600E and MSI are classified as tier IA targets, corresponding to the presence of a matching targeted drug that has shown clinically meaningful improvement in survival, while *ERBB2* amplification is classified as a tier IIA target, indicating a matching targeted therapy associated with response but without available survival outcomes.^4^^,^^6^ Without comprehensive testing, patients risk receiving suboptimal treatment, resulting in poorer outcomes, and may also suffer from complications associated with unsuitable therapies. Specifically, patients with *KRAS, NRAS,* or *BRAF* mutations and/or *ERBB2* amplifications do not benefit from, and indeed may be harmed by, anti-EGFR therapy,^1^, ^2^, ^3^ whereas patients with *BRAF* V600E mutations, *ERBB2* amplifications, and MSI are candidates for targeted therapy with BRAF inhibitors, HER2-targeted therapies, and immunotherapy, respectively.^1^, ^2^, ^3^, ^4^^,^^7^, ^8^, ^9^

Despite guideline recommendations, incomplete genotyping is rampant and often results in unsuitable treatment, leading to lack of benefit and premature disease progression.^3^ A retrospective analysis of 1497 patients found that only 52% of patients with mCRC were tested for *KRAS*, 38% for *NRAS*, 43% for *BRAF*, and 51% for MSI.^3^ More tellingly, 72% of patients receiving anti-EGFR therapy did not have guideline-recommended genotyping performed prior to commencing therapy, and therefore were at risk of receiving suboptimal or inappropriate treatment.^3^

A number of factors contribute to this undergenotyping, including use of sequential single-biomarker testing strategies, lack of available tissue for testing (estimated to affect 25% of patients with mCRC^3^^,^^10^), and failure of tissue-based tests. Moreover, the lengthy time associated with typical tissue-based genotyping can lead to patients beginning therapy before biomarker results are available, precluding their use in therapy selection.

Circulating tumor DNA (ctDNA) testing has emerged as an alternative to standard of care (SOC) tissue testing that addresses each of these barriers and is associated with improvements in the identification of candidates for recommended targeted therapies and patient outcomes in both clinical trials and real-world clinical practice.^10^, ^11^, ^12^, ^13^, ^14^, ^15^, ^16^, ^17^, ^18^, ^19^ Indeed, ctDNA testing has been reported to more than double the rate of targeted therapy delivery relative to tissue testing in multiple settings, primarily due to the ease of sample access, speed of result delivery, and low test failure rate.^14^, ^15^, ^16^, ^17^ A high degree of concordance has previously been demonstrated between tissue and ctDNA when testing for *RAS* mutations in patients with mCRC with BEAMing and NGS hotspot testing.^18^^,^^19^ However, no studies to date have examined the effectiveness of ctDNA testing in patients with 1L mCRC, or the value of comprehensive ctDNA testing relative to more limited panels.

In this study we evaluated the real-world performance and feasibility of both comprehensive and limited ctDNA testing relative to SOC tissue testing in patients newly diagnosed with mCRC. We report the ability of each test to identify unselected patients and turnaround time (TAT) in real-world clinical practice. Our findings demonstrate that the addition of ctDNA testing to clinical practice improves the diagnostic SOC in the treatment of patients with mCRC.

## Methods

### Clinical enrollment

Ethics Committee approval was granted by ‘Comite de Etica de la Investigacion Provincial de Malaga’ on 22 March 2018. A total of 158 patients with previously untreated mCRC were enrolled across three medical centers in Malaga, Spain. All patients were informed and consented in accordance with local regulations. Of these, 155 patients were determined to meet all study inclusion criteria and were included in the final analysis. All patient care was conducted according to the treating physician’s discretion, and all clinical information was obtained from the medical records of that care.

### Sample testing

Tissue testing was done according to the treating physician’s discretion following the SOC at the enrolling institution using real-time PCR with Idylla *KRAS* Mutation Test and Idylla *NRAS*-*BRAF* Mutation Test (Biocartis Inc.) in all cases, except three samples, in which real-time PCR with Therascreen *KRAS* RGQ PCR and pyrosequencing with *RAS* extension and *BRAF* Pyro Kit (Qiagen Inc.) were carried out. Mismatch repair (MMR) status was assessed with immunohistochemistry (for simplification the term MSI is used throughout the text to refer to both MMR and MSI testing). Comprehensive ctDNA testing was performed using Guardant360 (Guardant Health, Inc.) as previously described.^20^ Limited panel ctDNA testing was performed using the OncoBEAM *RAS* and *BRAF* CRC tests (Sysmex, Inc.) according to the manufacturer’s instructions for use.^21^ Both ctDNA tests were performed on blood samples drawn concurrently before the initiation of any anti-cancer treatment for metastatic disease.

### Data analysis

Biomarkers eligible for analysis were taken from the NCCN and ESMO guidelines, including *KRAS* single-nucleotide variants (SNVs; exons 2-4), *NRAS* SNVs (exons 2-4), *BRAF* V600E, *ERBB2* amplifications, and MSI status. *NTRK* testing was excluded from the analysis due to its introduction to practice guidelines after study enrollment. Biomarker discovery rate was defined as the proportion of total patients positive for at least one guideline-recommended biomarker. Test TAT was calculated from the date of sample acquisition to the date of results report. Noninferiority was assessed as previously described.^13^ GraphPad Prism version 8 was used to perform statistical analyses indicated in the text.

## Results

### Patient accountability and characteristics

**Figure 1:**
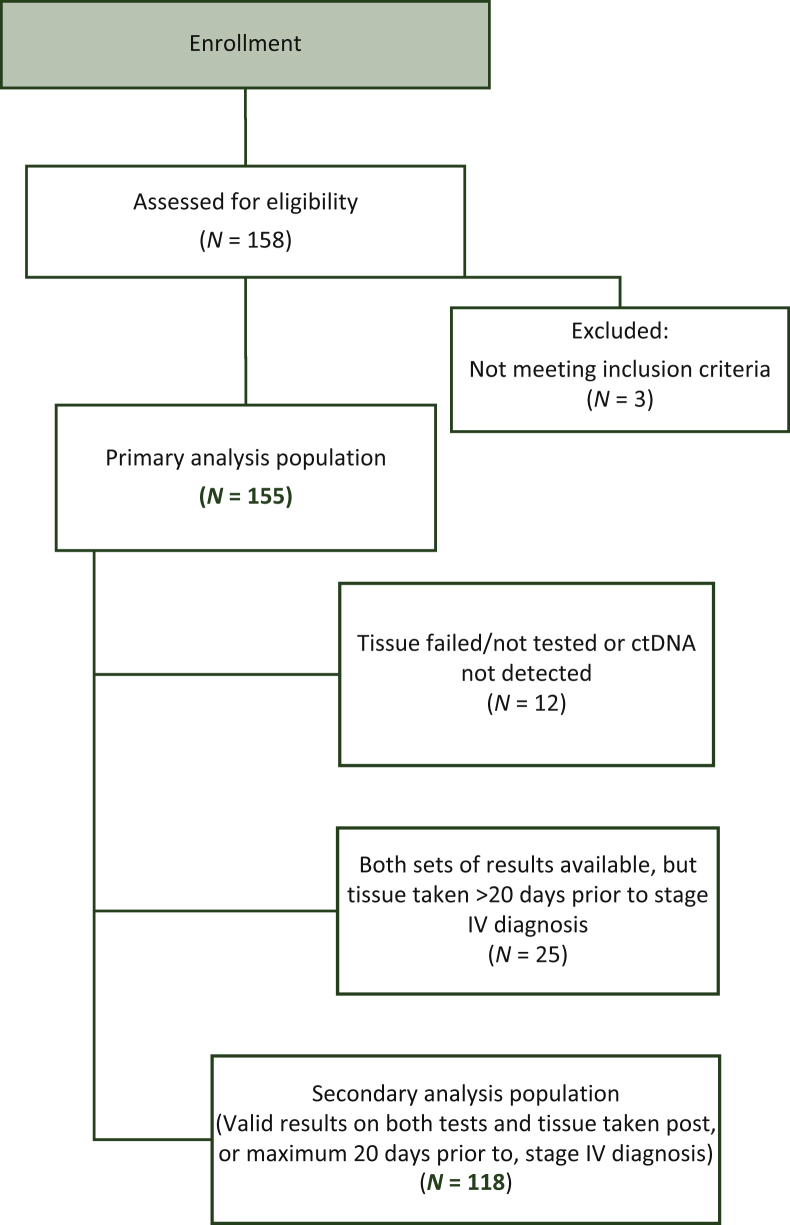
**Patient accountability.** ctDNA, circulating tumor DNA.

Of the 158 patients consented, 2 failed study inclusion criteria on screening and 1 was later found to have started 1L therapy prior to enrollment, leaving a total of 155 patients eligible for the primary analysis (Figure 1). ctDNA was detected in 98.1% (152/155) of patients. Tissue failed or was not tested in eight patients. Tissue testing was performed on newly taken biopsy specimens in 76.1% of patients, whereas 23.9% utilized archival resection specimens (Supplementary Table S1, available at https://doi.org/10.1016/j.esmoop.2022.100481). In 25 (16.1%) patients with valid results on both ctDNA and SOC testing, an archival tissue sample utilized for testing was found to have been collected at the patient’s initial CRC diagnosis at an earlier disease stage, prior to their progression to mCRC. In total, 118 (76.1%) patients had valid test results on both ctDNA and SOC tissue testing on tissue collected after, or up to 20 days before, diagnosis of stage IV disease (Figure 1). Demographics and baseline clinical characteristics were typical of the enrolling practices (Supplementary Table S1, available at https://doi.org/10.1016/j.esmoop.2022.100481).

### Biomarker discovery rate

**Figure 2:**
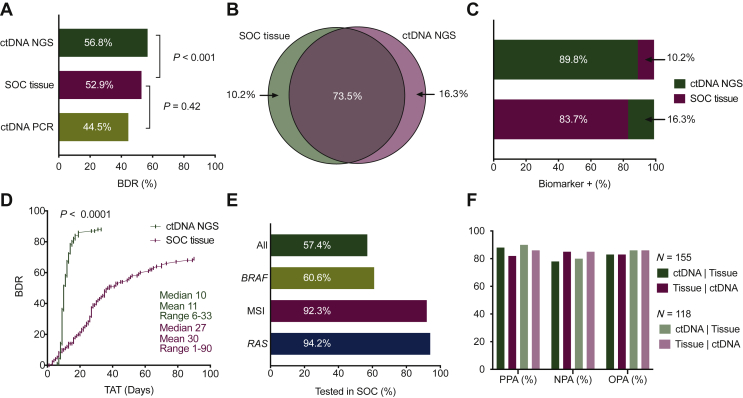
**Biomarker discovery rate and turnaround time (TAT).** (A) Biomarker discovery rate. Percent of total study patients with at least one guideline-recommended biomarker. (B) Relationship of patients positive by SOC tissue and comprehensive ctDNA testing. (C) Hypothetical sequencing of both orders of serial testing. Percent is out of total biomarker-positive patients identified. (D) Cumulative biomarker discovery rate as a function of time from sample collection to test result. (E) SOC tissue genotyping completion rate by biomarker. *BRAF* is *BRAF* V600E. ‘All’ includes *RAS*, *BRAF* V600E, and MSI status. (F) Clinical performance summary statistics for each testing method using the other as the comparator. Primary analysis population, all 155 patients on study; secondary analysis population, patients with valid results on both tests and tissue taken at stage IV. BDR, biomarker discovery rate; ctDNA, circulating tumor DNA; MSI, microsatellite instability; NGS, next-generation sequencing; NPA, negative percent agreement; OPA, overall percent agreement; PPA, positive percent agreement; SOC, standard of care.

For the primary endpoint of biomarker discovery, at least one guideline-recommended biomarker was identified by SOC tissue testing in 82 of the 155 eligible patients in the primary analysis population, in 88 patients by comprehensive ctDNA testing with Guardant360 (52.9% versus 56.8%, respectively, noninferiority demonstrated down to α = 0.005, 99% confidence interval 0.912-1.278; Figure 2A), and in 69 by limited ctDNA PCR testing (52.9% versus 44.5%, noninferiority rejected at α = 0.05, 90% confidence interval 0.736-0.952; Figure 2A). These results confirm noninferiority of comprehensive ctDNA NGS versus SOC tissue genotyping, but do not support noninferiority of limited ctDNA testing. Based on this result, further analyses were conducted to investigate the patient populations identified by tissue and comprehensive ctDNA NGS testing only.

In the primary analysis population of 155 patients, collectively SOC tissue and comprehensive ctDNA NGS testing identified 98 (63.2%) patients positive for at least one mCRC guideline-recommended biomarker, of which 83.7% (82/98) were positive by tissue and 89.8% (88/98) were identified with ctDNA testing. In the collectively identified 98 biomarker-positive patients, 73.5% (72/98) were positive for at least one guideline-recommended biomarker on both tissue and ctDNA, 10.2% (10/98) were positive on tissue only, and 16.3% (16/98) were positive on ctDNA only (Table 1 and Figure 2B). Of the 26 (16.8%) patients positive on only one test, 23.1% (6/26) lacked valid results from the second test; of these, 4 were successfully genotyped by ctDNA but not tissue, and 2 were successfully genotyped by tissue but not ctDNA.

**Table 1:** Guideline-recommended biomarker status by comprehensive ctDNA and SOC tissue testing

**Primary analysis population (*N* = 155)**^a^							
		Tissue				ctDNA to tissue, %	Tissue to ctDNA, %
		Positive	Negative	Total	PPA	87.8	81.8
ctDNA	Positive	72	16	88	PPV	81.8	87.8
	Negative	10	57	67	NPA	78.1	85.1
	Total	82	73	155	NPV	85.1	78.1
					OPA	83.2	83.2

**Secondary analysis population (*N* = 118)**^b^							
		Tissue				ctDNA to tissue, %	Tissue to ctDNA, %
		Positive	Negative	Total	PPA	89.6	85.7
ctDNA	Positive	60	10	70	PPV	85.7	89.6
	Negative	7	41	48	NPA	80.4	85.4
	Total	67	51	118	NPV	85.4	80.4
					OPA	85.6	85.6

Biomarkers included are *KRAS* SNVs, *NRAS* SNVs, *BRAF* V600E, MSI-H, and *ERBB2* CNVs.

ctDNA, circulating tumor DNA; NPA, negative percent agreement; MSI, microsatellite instability; NPV, negative predictive value; OPA, overall percent agreement; PPA, positive percent agreement; PPV, positive predictive value; SNV, single-nucleotide variant; SOC, standard of care.

a Samples that were negative, in which testing failed, which had no detectable tumor, or were not assessed for all biomarkers of interest are classified as ‘negative’.

b Secondary analysis population excludes samples in which testing failed, which had no detectable tumor, were not assessed for biomarkers of interest (*N* = 12), or samples where tissue was taken >20 days prior to stage IV diagnosis (*N* = 25).

### Test order and turnaround time

To assess the optimal order of tissue and comprehensive ctDNA testing, we compared the incremental addition of each to the other (Figure 2C). When using comprehensive ctDNA first, followed by reflex testing with tissue for all patients in whom a biomarker was not identified (ctDNA testing negative), 89.8% (88/98) of all biomarker-positive patients were identified with the test, with tissue testing adding an incremental 10.2% (10/98) upon reflex testing. Using tissue first identified 83.7% (82/98), with an incremental addition of 16.3% (16/98) from comprehensive ctDNA testing. Comprehensive ctDNA identified a modestly greater percentage of patients in the first round.

In this study, median TAT from sample collection to result for comprehensive ctDNA testing was faster than tissue (median 10 versus 27 days; range 6-33 versus 1-90 days; *P* < 0.0001), which, when combined with a modestly higher absolute patient identification rate shown in the preceding text, resulted in an acceleration of overall patient identification [51/98 (52.0%) patients for comprehensive ctDNA versus 10/98 (10.2%) patients for tissue, *P* < 0.0001, at 10 days; 87/98 (88.8%) patients for comprehensive ctDNA versus 37/98 (37.8%) patients for tissue, *P* < 0.0001, at 27 days; Figure 2D]. Importantly, TAT from time of sample arrival in the laboratory to issuing of results was similar for ctDNA NGS testing and SOC tissue (Supplementary Figure S1, available at https://doi.org/10.1016/j.esmoop.2022.100481), indicating that the time required to retrieve the tissue specimen was a significant factor contributing to TAT.

Recommended genotyping for *KRAS*, *NRAS*, *BRAF* V600E, and MSI (not including *ERBB2* amplification and *NTRK* fusion as these were not recommended for CRC in professional guidelines and not tested on tissue at the time of patient enrollment) was completed by tissue testing in 57.4% (89/155) of patients, *RAS* and *BRAF* V600E testing was completed in 60.6% (94/155), and *RAS* testing alone was completed in 94.2% (146/155). In 5.2% (8/155) of patients, no biomarkers were tested due to insufficient tissue quantity, while 1 additional patient was tested only for MSI (Figure 2E).

### Test concordance

In the primary analysis population, overall percent agreement (OPA) for comprehensive ctDNA relative to SOC tissue testing was 83.2% (129/155), with a positive percent agreement (PPA) of 87.8% (72/82) and negative percent agreement (NPA) of 78.1% (57/73). When excluding patients with archival tissue collected >20 days prior to diagnosis of stage IV disease and patients failing either test, OPA of ctDNA NGS relative to SOC tissue testing increased to 85.6% (101/118), with a PPA of 89.6% (60/67) and an NPA of 80.4% (41/51; Table 1 and Figure 2F). OPA, PPA, and NPA for SOC tissue testing relative to comprehensive ctDNA were similar, 83.2% (129/155), 81.8% (72/88), and 85.1% (57/67), respectively, in the primary analysis population, and 85.6% (101/118), 85.7% (60/70), and 85.4% (41/48), respectively, in the secondary analysis population (Table 1 and Figure 2F).

**Figure 3:**
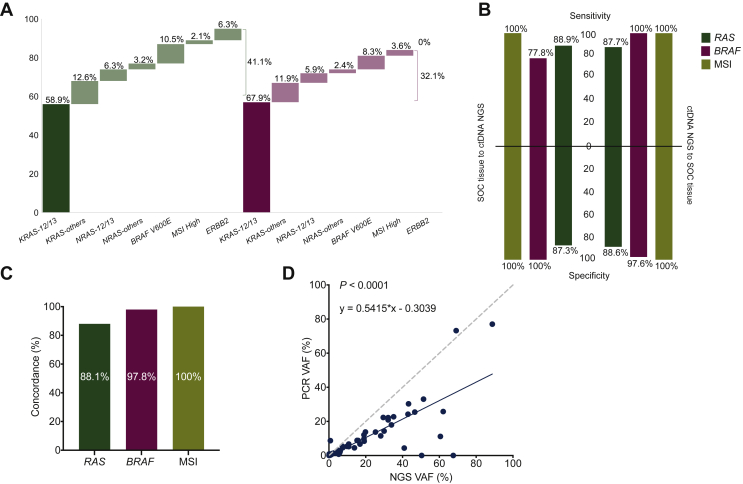
**Biomarker identity and concordance between comprehensive ctDNA and SOC tissue testing.** (A) Biomarkers identified by comprehensive ctDNA and SOC tissue testing. (B) Clinical performance summary statistics for each test method using the other as the comparator for each biomarker. *BRAF* is *BRAF* V600E. (C) Concordance between test methods per biomarker. (D) Correlation between variant allelic fractions (VAFs) as determined by comprehensive ctDNA NGS and limited ctDNA PCR testing for *KRAS*.ctDNA, circulating tumor DNA; NGS, next-generation sequencing; SOC, standard of care.

A total of 95 guideline-recommended biomarkers were identified by comprehensive ctDNA testing in 88 patients. The seven patients with more than one biomarker mostly comprised complex *RAS-*mutant alleles but also included one patient with co-occurring *ERBB2* amplification and *KRAS* mutation. SNVs in *KRAS* codons 12 and 13 comprised 58.9% (56/95) of all biomarkers identified, with the remainder being distributed between noncanonical activating *RAS* mutations (21/95, 22.1%), *BRAF* V600E mutations (10/95, 10.5%), MSI (2/95, 2.1%), and *ERBB2* amplifications (6/95, 6.3%; Figure 3A).

On tissue, 84 guideline-recommended biomarkers were identified in 82 biomarker-positive patients. SNVs in *KRAS* codons 12 and 13 comprised 67.9% (57/84) of all biomarkers identified, similar to the prevalence on ctDNA, with the remainder being distributed between noncanonical activating *RAS* mutations (17/84, 20.2%), *BRAF* V600E mutations (7/84, 8.3%), and MSI (3/84, 3.6%; Figure 3A). Testing for *ERBB2* amplification was not performed as part of tissue SOC.

Concordance between tissue and comprehensive ctDNA testing was high on a patient level; however, the data in the preceding text demonstrate that this can be influenced by multiple nonanalytical factors, including sample availability, and do not necessarily indicate concordance for individual biomarkers. As such, we investigated the concordance between both testing modalities for each biomarker assessed. Using tissue testing as the comparator, comprehensive ctDNA testing demonstrated high sensitivity, specificity, and overall concordance for each biomarker individually, with overall concordance ranging from 88.1% to 100% and sensitivity from 87.7% to 100%. As expected, similar findings were observed for tissue using ctDNA as the comparator (Supplementary Table S2, available at https://doi.org/10.1016/j.esmoop.2022.100481; Figure 3B and C). Concordance for *RAS* between comprehensive ctDNA and targeted *RAS* ctDNA PCR was 88.5% (116/131), with sensitivity of 84.6% (55/65) and specificity of 92.4% (61/66), and concordance between SOC tissue and targeted *RAS* ctDNA PCR was 84.8% (106/125), with sensitivity of 80.6% (50/62) and specificity of 88.9% (56/63). When comparing reported allelic fractions by simple linear regression, both the comprehensive and limited ctDNA tests correlated well for most samples (*P* < 0.0001), although the BEAMing technology used in the limited ctDNA panel demonstrated a lower molecule recovery than with NGS used in the comprehensive ctDNA test, as demonstrated by the slope of 0.5415 (Figure 3D), and susceptibility to allele drop out, potentially due to coalterations in probe-binding sites, as demonstrated by rare samples with high allelic fraction by NGS but low allelic fraction or no detection by BEAMing (Figure 3D).

### Clinical correlates of ctDNA

While comprehensive ctDNA testing demonstrated high concordance with tissue testing, 6.5% (10/155) of patients were positive by tissue testing but were negative (*N* = 8) or lacked results (*N* = 2) on comprehensive ctDNA testing. As such, it is critical to identify in which patients’ ctDNA might miss actionable biomarkers. To this end, we investigated various clinical features to identify which might inform as to the risk of negative ctDNA results.

**Figure 4:**
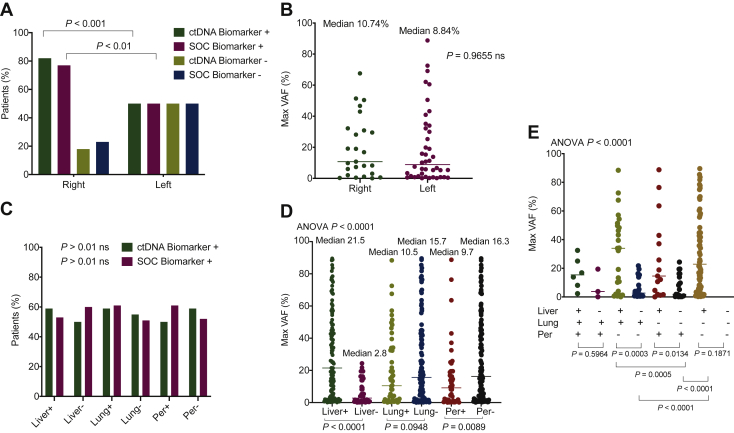
**Clinical correlates of ctDNA.** (A) Biomarker discovery rate for tissue and comprehensive ctDNA testing by primary tumor location. (B) Maximum variant allelic fraction (VAF) by primary tumor location. (C) Biomarker prevalence by metastases in liver, lung, and peritoneum. (D) Maximum VAF by presence versus absence of metastases at site. (E) Maximum VAF by metastatic site with all metastatic site combinations represented. There were no patients without metastasis in at least one of the aforementioned sites.ctDNA, circulating tumor DNA.

We first examined whether the anatomical location of the primary tumor might influence ctDNA detection. As previously reported, the anatomic location of the primary tumor influenced biomarker prevalence, particularly for *RAS* mutations; however, within anatomic locations, similar prevalence was observed using both ctDNA and SOC tissue testing (Figure 4A). Maximum variant allelic fraction (VAF) observed by ctDNA analysis, which is a primary determinant of ctDNA–tissue test concordance, was similarly unaffected by anatomic localization of the primary tumor (Figure 4B). By contrast, location of metastatic disease was highly correlated with maximum VAF, with the presence of liver metastases specifically correlated with higher ctDNA levels independent of metastatic involvement of other sites with a median maximum VAF of 21.5% in patients with the presence of liver metastasis and 2.8% in patients without metastasis to the liver (*P* < 0.0001). The most significant difference in maximum VAF was seen between patients with only liver metastasis and those with metastasis only to the lung or only to the peritoneum (Figure 4D and E). Despite this strong correlation, no significant differences were seen in biomarker discovery between metastatic sites (Figure 4C), perhaps suggesting that the ctDNA fraction, while lower in patients without liver metastases, is still sufficient for adequate genotyping in all patients. This hypothesis is supported by the observation that only 3 of the 155 patients eligible for evaluation lacked detectable ctDNA. In patients with unresected versus resected primary tumor the median number of alterations identified was 6 versus 5, respectively, and the median maximum VAF was 19.85% versus 8.20%, respectively; however, the *P* value was not statistically significant. In patients with liver metastases versus no liver metastases, the OPA for comprehensive ctDNA testing relative to SOC tissue was 82.2% (88/107) versus 85.4% (41/48), with a PPA of 92.5% (49/53) versus 79.3% (23/29) and an NPA of 72.2% (39/54) versus 94.7% (18/19) in liver-positive versus liver-negative patients, respectively.

## Discussion

In this prospective study, we demonstrate that comprehensive ctDNA testing identifies at least as many patients with guideline-recommended biomarkers as SOC tissue testing and does so in a shorter time. These findings are critical as the time required for genotyping results in current clinical practice is often infeasible, which leads to patients beginning treatment without the information necessary for optimal therapy decision making. In this study, comprehensive ctDNA analysis improved identification of patients with guideline-recommended actionable biomarkers relative to SOC tissue testing in the 2 weeks after sample collection, which is a key period when decisions regarding treatment are most often made. In addition, comprehensive ctDNA testing also obviated the need to coordinate multiple tests to achieve guideline-complete genotyping, resulting in nearly all (98.1%) patients receiving guideline-complete genotyping as compared with 57.4% with tissue testing, the importance of which is only growing as additional biomarkers enter clinical practice (e.g. *ERBB2* amplifications, *NTRK* fusions). In addition, when results were available for both tests, the concordance between each remained high (for each biomarker, sensitivity was 87.7%-100% and overall concordance 88.1%-100%), indicating that the overall patient population identified by both testing modalities is similar and thus can be treated similarly.

Despite rapid and relatively complete biomarker discovery, a minority of patients (6.5%) were identified by tissue but not comprehensive ctDNA testing, and a similar minority (10.3%) was identified by ctDNA but not tissue testing, suggesting that optimal patient identification would use both testing modalities simultaneously to maximize the opportunity for biomarker discovery. While such a dual-testing paradigm is likely optimal, it may not be economically feasible for both tests to be ordered on every patient. As such, we investigated the optimal order of tests used in serial. Because of the rapidity of result availability, we conclude that from a clinical perspective the optimal order of ctDNA and tissue testing is to use comprehensive ctDNA first, which based on our data here identified 89.8% of biomarker-positive patients with a median TAT of 10 days from test order. Tissue testing can then be ordered in those patients negative by ctDNA to identify the remaining 10.2% of biomarker-positive patients. Such test succession would result in more rapid and complete identification of biomarker-positive patients without incurring the full cost of concurrent testing.

In addition to accurately identifying patients that would have been positive by SOC tissue testing, ctDNA testing may offer a number of potential advantages that are not feasible when using tissue analysis. First is the immediate and facile access to the patient sample; ctDNA testing is performed on a peripheral blood draw, whereas tissue testing requires access to the tumor itself, which typically requires an invasive procedure. Beyond patient comfort, safety, and speed, peripheral blood draws ensure that genotyping results reflect the current genomic state of a patient’s tumor and overcome limitations of tissue heterogeneity. Our study found that 23.9% of tissue testing was performed on archival specimens, which may not accurately reflect the current genomic status of the tumor. The advantage of blood-based testing would be particularly pronounced when re-genotyping patients on progression to assess for targetable (e.g. *ERBB2* amplification) and/or resistance biomarkers (e.g. *RAS/RAF* mutations) acquired after therapy, where tissue would not typically be obtained for other purposes.

An important limitation of this study to highlight is that it is neither intended nor designed to examine the analytical concordance between tissue and ctDNA testing, most notably manifest in the facts that the two diagnostic platforms used distinct technologies, assessed distinct analytes, and that matched tissue-plasma samples were not collected. However, we feel this design is the most robust possible as our goal was not to assess tissue–ctDNA concordance but rather to compare two different clinical practices. As such, we do not draw conclusions regarding the analytical validity of the test methodologies, which have been reported elsewhere, but instead focus on the efficacy and feasibility of these different clinical practices.

Another important limitation of this study is that testing duration was captured using the date of sample acquisition and test result delivery and thus did not capture the duration of each individual step in this process, such as the time between sample acquisition and test order. The time elapsed from tissue collection to the ordering of molecular testing and to the subsequent arrival of the sample in laboratory contributed to the long median of 27 days seen in SOC. Longer TATs associated with tissue testing compared with ctDNA testing have previously been reported elsewhere and are not unique to the participating centres.^14^^,^^15^ We feel this measurement of duration is meaningful as it reflects the TAT as perceived by the treating physician and patient; however, it does limit further dissection of where differences occur between testing modalities, excludes examination of differences in delays associated with sample acquisition, and assumes that testing was conducted immediately after sample acquisition, prior to the initiation of any therapy. Each of these limitations may influence the effect that genotyping modalities may have in individual practices.

In conclusion, comprehensive ctDNA testing can provide accurate and complete genotyping results for patients newly diagnosed with mCRC more easily and rapidly than SOC tissue testing, allowing informed treatment choices without subjecting patients to delay. By using tissue testing in ctDNA-negative patients, such a strategy could improve time to treatment initiation without compromising biomarker discovery.
