# Supplementary figures and images for: Clinical utility of comprehensive circulating tumor DNA genotyping compared with standard of care tissue testing in patients with newly diagnosed metastatic colorectal cancer
Source: ESMO Open. 2022 May 4;7(3):100481. doi: 10.1016/j.esmoop.2022.100481 (PMC9271474; doi:10.1016/j.esmoop.2022.100481)

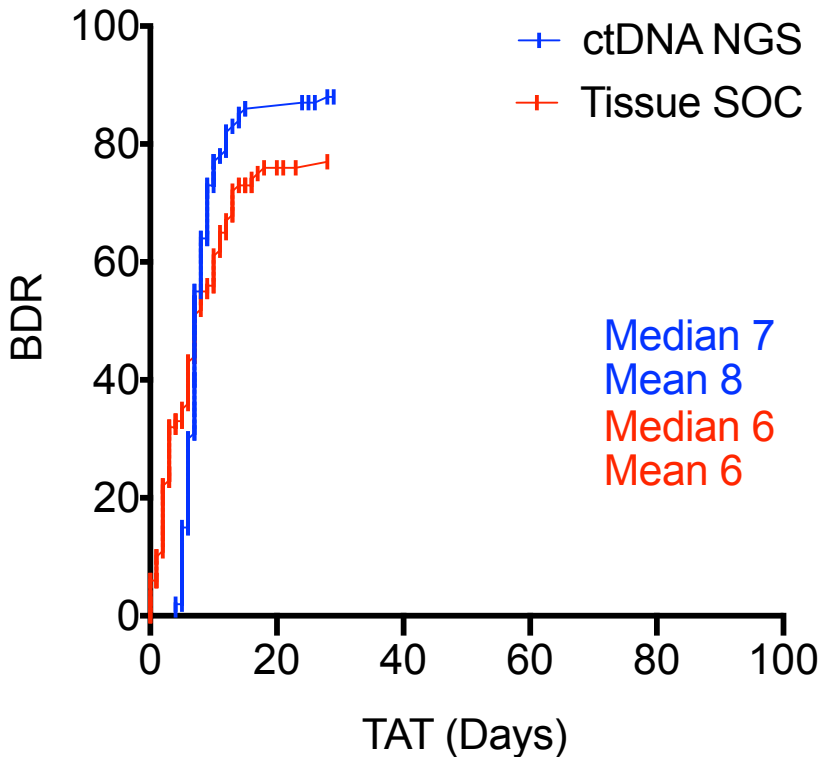

Supplement: Supplementary Fig 1 [file mmc1.pdf]
