# Supplementary material for: Clinical utility of comprehensive circulating tumor DNA genotyping compared with standard of care tissue testing in patients with newly diagnosed metastatic colorectal cancer
Source: ESMO Open. 2022 May 4;7(3):100481. doi: 10.1016/j.esmoop.2022.100481 (PMC9271474; doi:10.1016/j.esmoop.2022.100481)
Supplement: Supplementary Tables S1 and S2 [file mmc2.docx]

**Supplementary Table 1** **Patient demographics and baseline clinical characteristics.**

|  |  | **Number** |  | **Percentage (%)** |
| --- | --- | --- | --- | --- |
| Sex | Female | 65 |  | 41.9 |
|  | Male | 90 |  | 58.1 |
| Median age at enrollment (range) in years |  |  | 65 (31-87) |  |
| ECOG status at enrollment | 0 | 48 |  | 31.0 |
|  | 1 | 82 |  | 52.9 |
|  | 2 | 23 |  | 14.8 |
|  | 3 | 2 |  | 1.3 |
| Stage at CRC diagnosis | I | 5 |  | 3.2 |
|  | II | 8 |  | 5.2 |
|  | III | 18 |  | 11.6 |
|  | IV | 124 |  | 80.0 |
| History of prior chemotherapy for early-stage CRC | Yes | 20 |  | 12.9 |
|  | No | 11 |  | 7.1 |
|  | N/A (diagnosed stage IV) | 124 |  | 80.0 |
| CRC tumor pathology | Adenocarcinoma | 153 |  | 98.8 |
|  | Mucinous | 1 |  | 0.6 |
|  | Signet ring | 1 |  | 0.6 |
| Primary CRC tumor location | Right | 39 |  | 25.2 |
|  | Cecum | 15 |  | 9.7 |
|  | Ascending colon | 12 |  | 7.7 |
|  | Hepatic flexure | 11 |  | 7.1 |
|  | Transverse colon | 1 |  | 0.6 |
|  | Left | 115 |  | 74.2 |
|  | Splenic flexure | 13 |  | 8.4 |
|  | Descending colon | 3 |  | 2.0 |
|  | Sigmoid colon | 56 |  | 36.1 |
|  | Rectum | 43 |  | 27.7 |
|  | Unknown | 1 |  | 0.7% |
| Primary tumor resected | Yes | 83 |  | 53.5 |
|  | No | 72 |  | 46.5 |
| Source of tissue sample for SOC testing | Primary resection | 37 |  | 23.9 |
|  | Biopsy | 118 |  | 76.1 |
| Site of metastatic disease | Liver only | 54 |  | 34.8 |
|  | Lung only | 17 |  | 11.0 |
|  | Peritoneum only | 15 |  | 9.7 |
|  | Multiple sites | 69 |  | 44.5 |
| N/A: not applicable |  |  |  |  |

**Supplementary Table 2 Concordance between comprehensive ctDNA and SOC tissue testing by biomarker.**

| ***RAS (KRAS* and *NRAS)*** | | | **Tissue** |  |  | **ctDNA to Tissue** | **Tissue to ctDNA** |
| --- | --- | --- | --- | --- | --- | --- | --- |
|  |  | Positive | Negative | Total | Sensitivity | 87.7% | 88.9% |
|  | Positive | 64 | 8 | 72 | PPV | 88.9% | 87.7% |
| **ctDNA** | Negative | 9 | 62 | 71 | Specificity | 88.6% | 87.3% |
|  | Total | 73 | 70 | 143 | NPV | 87.3% | 88.6% |
|  |  |  |  |  | Concordance | 88.1% | 88.1% |
| ***BRAF* V600E** | | | **Tissue** |  |  | **ctDNA to Tissue** | **Tissue to ctDNA** |
|  |  | Positive | Negative | Total | Sensitivity | 100.0% | 77.8% |
|  | Positive | 7 | 2 | 9 | PPV | 77.8% | 100.0% |
| **ctDNA** | Negative | 0 | 82 | 82 | Specificity | 97.6% | 100.0% |
|  | Total | 7 | 84 | 91 | NPV | 100.0% | 97.6% |
|  |  |  |  |  | Concordance | 97.8% | 97.8% |
| **MSI** | | | **Tissue** |  |  | **ctDNA to Tissue** | **Tissue to ctDNA** |
|  |  | Positive | Negative | Total | Sensitivity | 100.0% | 100.0% |
|  | Positive | 2 | 0 | 2 | PPV | 100.0% | 100.0% |
| **ctDNA** | Negative | 0 | 105 | 105 | Specificity | 100.0% | 100.0% |
|  | Total | 2 | 105 | 107 | NPV | 100.0% | 100.0% |
|  |  |  |  |  | Concordance | 100.0% | 100.0% |

Patients not tested for the biomarker specified were excluded: 9 patients did not have *RAS* tested on tissue, 3 patients did not have ctDNA detected, 61 patients did not have *BRAF* V600E tested on tissue, 48 patients did not have MSI tested with tissue and/or ctDNA. PPV = Positive Predictive Value, NPV = Negative Predictive Value.
